# Alcohol Use and Mental Health among Older American Adults during the Early Months of the COVID-19 Pandemic

**DOI:** 10.3390/ijerph18084222

**Published:** 2021-04-16

**Authors:** Marisa R. Eastman, Jessica M. Finlay, Lindsay C. Kobayashi

**Affiliations:** 1 Center for Social Epidemiology and Population Health, Department of Epidemiology, University of Michigan School of Public Health, Ann Arbor, MI 48109, USA; meastma@umich.edu; 2 Survey Research Center, Institute for Social Research, University of Michigan School of Public Health, Ann Arbor, MI 48104, USA; jmfinlay@umich.edu

**Keywords:** older adults, alcohol use, COVID-19, mental health

## Abstract

Poor mental health associated with the COVID-19 pandemic may prompt the utilization of various coping behaviors, including alcohol use. We aimed to investigate the relationships between mental health symptomatology and self-reported changes in alcohol consumption at the onset of the pandemic. Data were from the nationwide COVID-19 Coping Study of US adults aged ≥55 in April and May 2020 (n = 6548). We used population-weighted multivariable-adjusted multi-nomial logistic regression models to estimate odds ratios (ORs) for the associations between mental health (of depression, anxiety, and loneliness, each) and self-reported increased alcohol consumption (vs. no change in consumption). One in ten adults (717/6548; 11%) reported an increase in their alcohol consumption in the past week compared to their usual pre-COVID-19 drinking. Mental health symptomatology was associated with increased drinking since the pandemic onset (depression: OR = 2.66, 95% CI: 1.99–3.56; anxiety: OR = 1.80, 95% CI: 1.34–2.42; loneliness: OR = 2.45, 95% CI: 1.83–3.28). Participants who screened positive for all three mental health outcomes were substantially more likely to report increased alcohol consumption since the onset of the pandemic (OR = 3.87, 95% CI: 2.52–5.96, vs. no mental health outcomes). This study demonstrates potentially harmful changes in alcohol intake among middle-to-older aged adults experiencing mental health symptomatology during the early months of the COVID-19 pandemic.

## 1. Introduction

The COVID-19 pandemic has disrupted the lives of people around the world, with the potential for substantial immediate and long-term mental health consequences [1,2,3]. In the United States (US), shelter-in-place and social distancing orders have challenged typical avenues of social support and engagement, and early studies have observed an increased prevalence of depression, anxiety, and loneliness at all ages in the US population [4,5,6,7,8,9]. Social isolation may be one of many factors contributing to poor mental health during the pandemic, and has previously been related to adverse mental health outcomes (including depression, anxiety, and loneliness) [1,10,11,12,13,14,15,16,17,18,19]. One study conducted among adults aged 18+ in the early months of the pandemic found the largest proportion of increased alcohol consumption among adults aged 18–39; however, adults aged 40 and over were much more likely to self-report increased alcohol use with comorbid adverse mental health symptomatology [19]. Behavioral coping mechanisms that older adults may engage in to deal with stress and social isolation (such as alcohol consumption) and their relationships with mental health during the COVID-19 pandemic remain largely unknown. This gap in evidence limits public health policy to mitigate the population mental health burden during and beyond the pandemic.

Alcohol consumption is a behavior commonly modified as a coping mechanism in response to life stressors, and has previously been understood according to the tension reduction hypothesis [20,21]. The tension reduction hypothesis suggests that alcohol consumption increases in response to cumulative and acute stress exposures in order to alleviate negative emotions [20,22,23]. Acute social isolation, health risks, and other life disruptions induced by the COVID-19 crisis may be such stressors, especially for middle-to-older aged adults who are at elevated risk for severe morbidity and mortality due to COVID-19 and may need to isolate more intensely than younger population groups [16,24,25,26,27,28,29,30]. While previous studies have produced mixed results supporting the tension reduction hypothesis, consistent evidence identifies a strong and bidirectional relationship between alcohol use and depression [22,23,31,32,33,34,35,36]. Further, previous studies suggest that exposure to stress or stressful stimuli are important motivators for increased alcohol intake [23,34,37,38,39,40,41,42,43,44]. However, it is not known how alcohol consumption may have changed among middle-to-older aged US adults during the COVID-19 pandemic.

In order to address this gap in evidence regarding pandemic-associated behavioral health outcomes, we analyzed data from a national sample of US adults aged ≥55 to estimate the relationships between self-reported changes in alcohol consumption with depression, anxiety, and loneliness at the onset of the COVID-19 pandemic. We hypothesized that individuals who screened positive for depression, anxiety, and loneliness would have a higher likelihood of increased alcohol consumption at the onset of the pandemic, compared to people who did not screen positive for any of these mental health indicators.

## 2. Materials and Methods

Data for this study were collected in the baseline questionnaire of the COVID-19 Coping Study from 2 April to 31 May 2020 [45]. The COVID-19 Coping Study is a national cohort study of mental health and well-being among US adults aged ≥55 during the COVID-19 pandemic, conducted by the University of Michigan. A total of 6938 US adults aged ≥55 from all 50 US states, the District of Columbia, and Puerto Rico completed the baseline questionnaire. The questionnaire was administered in an online format designed for computer, tablet, and smartphone interfaces through the University of Michigan’s Qualtrics survey software. The COVID-19 Coping Study was approved by the Health Sciences and Behavioral Sciences Institutional Review Board at the University of Michigan (HUM00179632), and all respondents provided online informed consent prior to starting the questionnaire. Participants eligible for this analysis were those who completed the baseline questionnaire of the COVID-19 Coping Study with non-missing data on alcohol consumption, mental health outcomes, and covariates. After all appropriate exclusions, our final analytic sample included 6635 individuals (96% of the total sample).

### 2.1. Measures

#### 2.1.1. Primary Outcome: Self-Reported Change in Alcohol Consumption in the Past Week

Participants were asked to indicate past-week changes in their alcohol consumption by answering the question: “over the past week, have any of your usual daily activities or behaviors changed?” in a section that “asks about your experiences and feelings related to the ongoing COVID-19 (coronavirus) pandemic.” This question asked study participants to indicate whether they were engaging in 15 specific activities or behaviors, including drinking alcohol “more than usual”, “about the same as usual”, “less than usual” or indicating “I don’t normally do this activity”. Our main outcome variable preserved these four categories to indicate self-reported changes in alcohol consumption over the past week, shortly after the onset of the COVID-19 pandemic.

#### 2.1.2. Primary Exposures: Depression, Anxiety, and Loneliness

Depression, anxiety, and loneliness were assessed in the questionnaire using validated research scales. Depression was assessed using the 8-item Center for Epidemiologic Studies depression scale (CES-D), adapted from the US Health and Retirement Study. The 8-item CES-D asks participants to indicate the presence of eight depressive symptoms “much of the time” over the past week (yes, no), and total scores of ≥3/8 indicated a positive screen for depression. Anxiety was assessed using the 5-item Beck Anxiety Inventory (BAI), adapted from the US Health and Retirement Study Psychosocial and Lifestyle Questionnaire [46]. The 5-item BAI asks participants to rate the frequency of anxiety symptoms in the past week using a 4-point Likert response scale ranging from 1 = “never” to 4 = “most of the time”. Total scores of ≥10/20 indicated a positive screen for anxiety, representing the upper 25th percentile of scores in this sample. Loneliness was assessed using the 3-item UCLA Loneliness Scale, which asks participants to report how often they felt they lacked companionship, felt left out, or isolated from others in the past week using a 3-item Likert response scale ranging from 1 = “hardly ever” to 3 = “often”. Total scores of ≥6/9 indicated a positive screen for loneliness, representing the upper 25th percentile of scores in this sample [47].

#### 2.1.3. Secondary Exposure: Mental Health Burden

The secondary exposure variable in this study was mental health burden, which was defined as the total number of positive mental health screening indicators (depression, anxiety, and loneliness) and had a range from 0 (reference) to 3.

### 2.2. Covariates

Covariates were collected in the baseline questionnaire and included: age (years), sex (male; female), race/ethnicity (non-Hispanic White; non-Hispanic Black; Hispanic/Latinx; East Asian, Native Hawaiian, and Pacific Islander; other), education (high school diploma/equivalent or less then high school; some college or 2-year Associate’s degree; 4-year college or university degree; post-graduate or professional degree), relationship status (never married; divorced/separated; widowed; married/in a relationship), pre-pandemic employment status (employed; retired; unemployed and seeking work; unable to work due to disability or health condition; homemaker/family caregiver), pre-pandemic weekly moderate-to-vigorous intensity physical activity (none; less than 30 min; 30 min to <1 h; 1 h to <1.5 h; 1.5 h to <2 h; 2 h to <2.5 h; 2.5 h or more), pre-pandemic weekly alcoholic beverages consumed (0 to 10+), smoking status (never smoked; former smoker; current smoker), having a friend or family member with symptoms of or diagnosed with COVID-19 (yes; no), history of physician-diagnosed hypertension, diabetes, heart disease, asthma, chronic obstructive pulmonary disease (COPD), cancer, or other limiting, long-standing health condition (yes; no for each), and degree of pre-pandemic social isolation (low; high). Social isolation was evaluated by five binary items according to the English Longitudinal Study of Ageing social isolation index: less than monthly contact with children, less than monthly contact with family, less than monthly contact with friends, less than monthly participation in a social organization or club, and, living alone [47]. High social isolation was defined as scoring ≥2/5 on the index [47]. Pre-pandemic alcohol consumption was assessed by asking participants, “before the COVID-19 (coronavirus) pandemic, how many drinks of alcohol did you usually have per week (e.g., glasses of wine, beer, or shots of spirits/hard liquor)?” A dropdown list of integers ranging from “0” to “10+” was provided for participants to indicate their answer.

### 2.3. Statistical Analysis

We generated population weights using data from the 2018 American Community Survey to ensure that our study sample is representative of the general US population aged ≥55 based on age, sex, race, ethnicity, education, marital status, and US census region of residence [45]. All estimates in this analysis incorporate these population weights. The characteristics of the population-weighted sample, overall and according to self-reported changes in alcohol consumption were described using univariate statistics. We specified multivariable-adjusted, population-weighted multi-nomial logistic regression models with a glogit function for categorical outcome data to estimate odds ratios (ORs) and 95% confidence intervals (CIs) for self-reported changes in alcohol consumption in the past week, according to anxiety, depression, loneliness, and total mental health burden as predictor variables [48]. Four separate models were estimated, one for each mental health predictor. The reference category of the alcohol outcome variable was “no change in usual drinking” in order to evaluate how each mental health predictor was related to changes in alcohol consumption, compared to those who maintained their typical consumption patterns. All models were adjusted for the confounders described in the previous section. Analyses were conducted using SAS 9.4 (Cary, NC, USA). All *p*-values were two-tailed and statistical significance was evaluated at the 0.05 level.

## 3. Results

The mean age (standard error; SE) of the population-weighted sample was 67.7 (0.2) years, and 53.5% (95% CI: 51.5–55.4%) of the sample was female (Table 1). Those who reported drinking more than usual in the past week at the onset of the COVID-19 pandemic also reported slightly drinking more frequently prior to the pandemic (mean: 4.7 drinks/week; SE: 0.2) than those who reported no change in their alcohol consumption (mean: 4.6 drinks/week; SE: 0.1) and those who reported drinking less than usual (mean: 3.4 drinks/week; SE: 0.2; Table 1). Approximately one in five adults (18.0%; 95% CI: 16.6–19.5%) reported a change in their alcohol consumption in the past week, of which 47% (636/1353) identified a decrease and 53% (717/1353) acknowledged an increase in their consumption.

Overall, 32.0% (95% CI: 30.3–33.8%) of the population-weighted sample screened positive for depression, 29.3% (95% CI: 27.5–31.1%) screened positive for anxiety, and 29.6% (95% CI: 27.9–31.4%) screened positive for high levels of loneliness. Among those who screened positive for depression, 13.3% (95% CI: 11.2–15.3%) reported consuming more alcohol in the past week than their usual pre-pandemic levels of consumption, and 11.4% (95% CI: 9.4–13.4%) indicated drinking less than usual (Table 2). Among those who screened positive for anxiety, 11.8% (95% CI: 9.8–13.8%) reported consuming more alcohol in the past week than their usual pre-pandemic levels of consumption, and 10.4% (95% CI: 8.5–12.4%) reported drinking less than usual (Table 2). Among those who were “high” in loneliness, 12.4% (95% CI: 10.2–14.5%) reported consuming more alcohol in the past week than their usual pre-pandemic levels of consumption, and 11.4% (95% CI: 9.2–13.6%) reported drinking less than usual (Table 2).

Table 3 presents results from population-weighted, multivariable-adjusted, multi-nomial logistic regression models predicting self-reported changes in alcohol consumption in the past week during the early COVID-19 pandemic, with “no change from usual pre-pandemic drinking levels” as the reference category. Individuals who screened positive for either depression, anxiety, or loneliness had greater odds of increased alcohol consumption, compared to those without any of the listed mental health indicators: OR = 2.65 (95% CI: 1.98–3.55, vs. no change from usual drinking) for depression, OR = 1.79 (95% CI: 1.33–2.41) for anxiety, and OR = 2.42 (95% CI: 1.81–3.25) for loneliness (Table 3). Those who screened positive for each of depression and loneliness had higher odds of drinking less than usual (depression: OR = 1.67; 95% CI: 1.23–2.25, vs. no change from usual drinking; loneliness: OR = 1.64; 95% CI: 1.21–2.24, vs. no change in usual drinking; Table 3).

Table 4 presents the count of mental health outcomes, overall and according to self-reported changes in alcohol consumption. Individuals who screened positive for no mental health outcomes (unweighted n = 3251) most frequently reported no change in their usual drinking (40.2%; 95% CI: 37.5–42.8%), while only 23.6% (95% CI: 18.8–28.5%) of individuals who screened positive for all three mental health outcomes (unweighted n = 729) reported no change in their usual drinking in the past week (Table 4). Those who screened positive for two (unweighted n = 1132) and three (unweighted n = 729) mental health outcomes most frequently reported drinking more than usual in the past week (13.1%; 95% CI: 10.4–15.8% for two outcomes, and 15.0%; 95% CI: 11.3–18.8% for three outcomes; Table 4).

Table 5 presents results from population-weighted, multi-variable multi-nomial logistic regression models predicting self-reported changes in alcohol consumption in the past week during the early COVID-19 pandemic, with no change from usual pre-pandemic drinking levels as the reference category. Those who screened positive for two mental health outcomes were more likely to report drinking more than usual (OR = 2.67; 95% CI: 1.86–3.83, vs. no change from usual drinking) as well as drinking less than usual (OR = 1.58; 95% CI: 1.09–2.31, vs. no change from usual drinking) in the past week, compared to those who screened positive for no mental health outcomes (Table 5). Similarly, those who screened positive for all three mental health outcomes had higher odds of reporting a decrease in alcohol consumption (OR = 2.06; 95% CI: 1.28, 3.30, vs. no change from usual drinking) as well as an increase in alcohol consumption (OR = 3.83; 95% CI: 2.48, 5.92, vs. no change from usual drinking; Table 5).

## 4. Discussion

In this national study of middle-aged and older U.S. adults, we observed a high prevalence of anxiety, depression, and loneliness alongside marked self-reported changes in alcohol consumption during the early months of the COVID-19 pandemic. The prevalence of anxiety, depression, and loneliness observed in our study is higher than pre-pandemic national levels; however, it is consistent with estimates from the National Poll on Healthy Aging and a mental health survey conducted by the CDC in June 2020 [4,49,50]. The specific etiology of these increases has been attributed broadly to pandemic-associated stressors; these will be discussed further based on insights provided by our results. We observed strong associations between experiencing depression, anxiety, or loneliness and increased alcohol consumption in the past week, with a dose–response relationship between overall mental health symptom burden and drinking either more or less in the past week than usual (before the COVID-19 pandemic). This research gives insight into how middle-aged and older US adults used alcohol during a period of vast uncertainty and stress, when most of the US was under shelter-in-place orders with rising COVID-19 cases and death counts [51].

### 4.1. Comparison to Existing Literature

We add to previous literature on mental health and alcohol use, finding that both increases and decreases in usual alcohol were common, and were often experienced alongside mental health symptomatology among middle-aged and older US adults during the early COVID-19 pandemic, a period of collective adversity. Evidence suggests that increases in alcohol consumption are concomitant with adverse mental health outcomes following large-scale traumatic exposures, such as a natural disaster, widespread economic assault, or acts of terrorism [37,38,39,40,41,43,44,52,53,54]. Taken together, these findings generally support the tension reduction hypothesis, indicating that alcohol consumption may be used to cope with negative emotions triggered by stress [20,22,23,42]. For example, studies conducted on the immediate and long-term aftermath of the terrorist attacks on the World Trade Center on 11 September 2011 (9/11) found that adults with high 9/11-exposure or high 9/11-PTSD were at an increased risk of subsequent binge drinking [38,39,52,55,56,57]. Although we were unable to assess the severity of exposure to the COVID-19 pandemic or binge drinking in our study, our results corroborate previous findings by showing high proportions of adverse mental health symptomatology and notable changes in alcohol consumption in the face of myriad stressors produced by the COVID-19 pandemic.

Furthermore, it is important to give attention to the unique context of the COVID-19 pandemic and consider its impact on the interpretation of our results. Unlike previous collective traumas, the COVID-19 pandemic has induced widespread social isolation, a necessary consequence of virus mitigation orders [1,11,16,27]. Previous research has shown that social isolation can lead to poor physical, cognitive, and mental health, as well as increases in alcohol use [58,59,60,61]. The physical social isolation experienced by many during the early months of the pandemic may be a common cause and likely explanation for the relationships we observed between mental health symptomatology and increased alcohol consumption.

The relationship between mental health symptomatology and the decreased alcohol consumption that we observed may be explained in the context of social and economic changes due to the COVID-19 pandemic. Profound and sudden economic or job losses may cause affected individuals to reallocate or decrease their spending, cutting out alcohol purchases [60]. Alcohol may have become unavailable in specific contexts due to closures of stores, restaurants, and bars, and limitations on the types of social activities where alcohol is often consumed may have also affected alcohol intake [60]. Some people may be less interested in consuming alcohol outside of social engagements [60]. Physical and mental health concerns posed by the risk of COVID-19 infection may also prompt decreased alcohol consumption [60]. Previous qualitative research conducted during the COVID-19 pandemic has cited concerns over amplifying negative mental health symptomatology through alcohol use, as well as desires to prioritize healthy lifestyle choices and decrease alcohol use to support the immune system as reasons for decreased alcohol consumption [62].

Our study took a novel approach in evaluating the accumulation of multiple mental health symptom burdens in relation to alcohol use. Previous literature is sparse in this area, except to acknowledge that alcohol use is a common behavior seen among individuals with mood disorders [34,35,36,37,63,64,65]. Our results suggest that middle-aged and older adults with multiple mental health symptomatologies are more likely to increase their alcohol consumption during periods of collective stress associated with the COVID-19 pandemic. One explanation for this finding could be that people with multiple mental health symptomatologies have a lower psychological resilience to stressors, causing them to employ different strategies for coping, such as alcohol use. These findings may be generalizable to other collective national traumas, including potential future pandemics, which is an important area for future research attention.

### 4.2. Strengths and Limitations

A limitation of this study is its cross-sectional nature, which prevents us from determining the causal direction of association between mental health and alcohol use. We did not have information on participants’ mental health prior to the pandemic. However, the prevalence of depression, anxiety, and loneliness that we observed is consistent with that found in nationally representative surveys, such as the National Poll on Healthy Aging and a mental health survey conducted by the CDC in June 2020 [4,49,50]. Furthermore, our results rely on the accuracy of self-reported data. Reluctance to honestly answer questions that may be perceived negatively is a limitation of many survey-based studies. The underreporting of alcohol use or mental health symptomatology may have led our estimates to underestimate the true magnitudes of these associations. This analysis did not include region or area of residence, such as urban versus rural location. Level of geographical isolation is frequently associated with access to services and social support [66], which may impact the relationship between mental health indicators and changes in coping behaviors, such as alcohol consumption. Finally, although we were able to obtain a large sample with representation from all 50 US states, the District of Columbia, and Puerto Rico, it is not a probability-based sample. Selection bias may be present if selection into the study was differential based on mental health and alcohol use, or according to any unmeasured factors that are uncorrelated with the sociodemographic variables used to weight our sample to the US general population. Finally, our results may not be generalizable to non-Internet users, if the associations under study differ in this population group relative to Internet users.

Our study has several strengths that bolster its contribution to knowledge of middle-aged and older adults’ mental health and alcohol use during the early months of the COVID-19 pandemic. We obtained rich social, economic, and behavioral data on a large sample size of English- and Spanish-speaking participants from all 50 US states and Puerto Rico. These data were collected during a unique period in time during the first wave of the pandemic when much of the US was under shelter-in-place orders and COVID-19 case and death counts were rapidly rising. We assessed anxiety, depression, and loneliness using research validated scales that are incorporated in other national longitudinal studies of aging (such as the US Health and Retirement Study), which increases the comparability of our findings to those of other studies. Future research may build on this study by incorporating longitudinal data to understand changes in the prevalence of mental health indicators and their impact on alcohol use among middle-aged and older U.S. adults throughout the COVID-19 pandemic and after.

## 5. Conclusions

In this large cross-sectional study of middle-aged and older US adults during the first wave of the COVID-19 pandemic, we found that changes in alcohol consumption since the start of the pandemic were associated with experiencing depression, anxiety, and loneliness. These findings draw attention to an unmet need for mental health and stress-coping resources. These results highlight the relationship between mental health, stress exposure, and alcohol use, which may serve as a foundation to identify individuals at-risk for developing problematic alcohol use and tailoring interventions to better help older adults cope with stress and isolation.

## Figures and Tables

**Table 1:** Population-weighted characteristics of the sample, COVID-19 coping study, United States, April–May 2020, N = 6548.

Covariates	Overall (N = 6548)	Change in Alcohol Consumption in the Past Week, Compared to Pre-COVID-19 Drinking			
		Don’t Usually Do This Activity (n = 2717)	Drinking Less than Usual (n = 636)	No Change in Drinking (n = 2478)	Drinking More than Usual (n = 717)
	% (95% CI)	% (95% CI)	% (95% CI)	% (95% CI)	% (95% CI)
Age, mean (SE), years	67.7 (0.2)	68.5 (0.4)	66.2 (0.6)	67.7 (0.3)	64.7 (0.5)
Sex					
Female	53.5 (51.5, 55.4)	60.5 (57.6, 63.4)	46.5 (40.3, 52.7)	44.6 (41.5, 47.7)	58.9 (53.3, 64.5)
Race/Ethnicity					
Non-Hispanic White	73.6 (71.7, 75.5)	73.9 (71.1, 76.7)	62.9 (55.9, 69.9)	75.2 (72.0, 78.3)	78.2 (72.4, 84.0)
Non-Hispanic Black	10.3 (8.9, 11.7)	10.6 (8.6, 12.7)	15.5 (10.2, 20.9)	9.0 (6.7, 11.3)	7.9 (3.9, 12.0)
Hispanic/Latinx	9.3 (7.9, 10.7)	7.8 (6.0, 9.7)	15.8 (9.0, 22.5)	9.2 (7.1, 11.4)	10.2 (5.6, 14.8)
East Asian/Native Hawaiian/Pacific Islander	4.4 (3.5, 5.4)	5.3 (3.7, 6.9)	3.4 (1.4, 5.5)	4.1 (2.6, 5.7)	2.0 (0.5, 3.6)
Other	2.3 (1.9, 2.8)	2.4 (1.7, 3.1)	2.4 (1.0, 3.8)	2.4 (1.6, 3.3)	1.6 (0.3, 3.0)
Education					
High School diploma or less	44.0 (41.9, 46.0)	50.7 (47.8, 53.6)	41.3 (34.4, 48.2)	38.3 (34.9, 41.7)	32.3 (25.9, 38.6)
Some college or 2 year associate degree	27.7 (26.0, 29.3)	26.8 (24.4, 29.2)	28.1 (22.7, 33.5)	28.7 (25.9, 31.4)	28.0 (23.0, 33.0)
4-year college or university degree	16.3 (15.3, 17.3)	13.4 (12.0, 14.7)	18.4 (14.9, 21.8)	18.2 (16.5, 20.0)	22.6 (18.7, 26.6)
Postgraduate or professional degree	12.1 (11.3, 12.8)	9.1 (8.2, 10.0)	12.2 (9.8, 14.6)	14.8 (13.4, 16.1)	17.1 (14.4, 19.1)
Relationship status					
Single, never married	8.2 (7.2, 9.2)	9.0 (7.5, 10.6)	7.7 (4.6, 10.9)	6.9 (5.2, 8.5)	9.6 (6.3, 12.9)
Single, divorced/separated	17.9 (16.4, 19.5)	18.8 (16.6, 21.0)	22.7 (16.5, 28.8)	15.6 (13.1, 18.1)	16.9 (12.0, 21.8)
Single, widowed	14.6 (13.1, 16.2)	17.1 (14.6, 19.7)	10.3 (6.0, 14.6)	12.6 (10.3, 14.8)	14.0 (9.1, 18.9)
Married or in a relationship	59.2 (57.3, 61.2)	55.0 (52.0, 57.9)	59.3 (52.7, 65.8)	65.0 (61.8, 68.1)	59.5 (53.6, 65.4)
Pre-COVID Employment status					
Employed	31.4 (29.7, 33.0)	26.2 (23.9, 28.5)	36.1 (30.4, 41.8)	33.8 (30.9, 36.6)	45.4 (39.8, 51.0)
Retired	52.7 (50.8, 54.6)	53.8 (50.9, 56.7)	51.8 (45.5, 58.2)	54.2 (51.0, 57.3)	41.4 (35.6, 47.1)
Unemployed and seeking work	8.1 (6.9, 9.2)	11.1 (9.1, 13.1)	4.0 (1.4, 6.6)	5.4 (3.7, 7.2)	6.5 (3.4, 9.5)
Unable to work	2.8 (2.0, 3.6)	2.6 (1.4, 3.8)	4.4 (1.8, 7.0)	2.8 (1.2, 4.3)	2.3 (0.4, 4.1)
Homemaker/Family caregiver ^a^	5.1 (4.1, 6.1)	6.4 (4.7, 8.1)	3.7 (1.5, 5.8)	3.8 (2.4, 5.3)	4.4 (1.7, 7.2)
Pre-COVID weekly physical activity					
None	16.0 (14.4, 17.6)	17.2 (14.9, 19.5)	16.9 (10.2, 23.6)	15.3 (12.7, 17.8)	11.3 (6.7, 15.9)
<30 min	16.5 (15.0, 18.0)	19.9 (17.4, 22.3)	17.4 (12.4, 22.3)	12.0 (10.0, 14.0)	15.2 (10.6, 19.7)
30 min–<1 h	14.1 (12.7, 15.5)	14.7 (12.6, 16.8)	19.8 (14.8, 24.7)	12.6 (10.3, 14.8)	10.6 (7.3, 13.9)
1 h–<1.5 h	9.4 (8.3, 10.5)	9.3 (7.7, 10.9)	7.1 (4.6, 9.6)	9.5 (7.5, 11.5)	11.9 (8.3, 15.5)
1.5 h–<2 h	7.9 (6.9, 9.0)	6.4 (4.9, 7.9)	8.0 (5.1, 11.0)	9.8 (7.8, 11.7)	8.7 (5.7, 11.6)
2 h–<2.5 h	7.4 (6.4, 8.3)	6.5 (5.2, 7.9)	8.8 (5.6, 12.1)	8.0 (6.4, 9.6)	7.7 (4.5, 10.9)
2.5 + h	28.7 (27.1, 30.3)	26.0 (23.6, 28.4)	22.0 (17.9, 26.2)	32.8 (30.0, 35.6)	34.6 (29.5, 39.7)
Pre-COVID alcoholic drinks/week, mean (SE)	2.4 (0.06)	0.1 (0.01)	3.4 (0.2)	4.6 (0.1)	4.7 (0.2)
Smoking Status					
Never Smoked	47.8 (45.9, 49.7)	51.3 (48.3, 54.2)	46.8 (40.4, 53.2)	44.6 (41.6, 47.7)	42.8 (37.3, 48.2)
Former Smoker	38.4 (36.5, 40.3)	35.6 (32.7, 38.4)	38.9 (32.9, 45.0)	41.8 (38.7, 44.9)	39.5 (34.0, 44.9)
Current Smoker	13.8 (12.3, 15.3)	13.2 (11.1, 15.3)	14.3 (9.1, 19.4)	13.6 (10.8, 16.3)	17.8 (12.1, 23.5)
Family member or friend diagnosed with COVID or had COVID-like symptoms					
Yes	16.1 (14.8, 17.3)	15.6 (13.6, 17.5)	18.7 (14.1, 23.2)	14.7 (12.7, 16.6)	21.6 (17.2, 25.9)
History of chronic conditions					
Hypertension	52.0 (50.1, 53.9)	55.0 (52.1, 57.9)	45.9 (39.4, 52.4)	49.4 (46.3, 52.6)	52.6 (47.0, 58.3)
Diabetes	17.1 (15.6, 18.6)	20.9 (18.5, 23.2)	16.7 (12.1, 21.2)	5.8 (11.4, 16.4)	9.2 (5.9, 12.6)
Heart Disease	9.7 (8.5, 10.8)	10.8 (8.8, 12.8)	11.2 (7.2, 15.2)	8.2 (6.6, 9.7)	7.4 (4.7, 10.2)
Asthma	9.8 (8.6, 11.0)	10.0 (8.0, 12.0)	9.1 (6.3, 11.9)	9.3 (7.2, 11.4)	11.6 (7.7, 15.5)
COPD	9.3 (7.9, 10.6)	10.9 (8.7, 13.1)	7.3 (4.3, 10.4)	6.9 (4.9, 8.8)	12.5 (7.3, 17.7)
Cancer	11.4 (10.3, 12.5)	11.7 (10.0, 13.3)	10.9 (6.8, 15.1)	11.6 (9.9, 13.4)	9.4 (6.4, 12.4)
Other	14.8 (13.5, 16.1)	17.5 (15.4, 19.5)	14.6 (10.0, 19.2)	11.5 (9.6, 13.5)	13.0 (9.4, 16.6)
High Social Isolation	43.1 (41.2, 45.1)	45.1 (42.2, 48.0)	47.5 (41.1, 54.0)	39.4 (36.2, 42.6)	42.0 (36.1, 47.9)

Abbreviations: SE, standard error; COPD, chronic obstructive pulmonary disease. ^a^ Participants who reported being a student (n = 2) are included in the homemaker/family caregiver category.

**Table 2:** Self-reported changes in alcohol consumption during the early months of the COVID-19 pandemic, according to mental health status, COVID-19 Coping Study, April-May 2020, N = 6548.

Mental Health Status	Don’t Usually Do This Activity (n = 2717)		Drinking Less than Usual (n = 636)		No Change in Drinking (n = 2478)		Drinking More than Usual (n = 717)	
	%	95% CI	%	95% CI	%	95% CI	%	95% CI
Depression								
No	47.0	44.6, 49.4	9.1	7.6, 10.6	38.1	35.9, 40.4	5.8	4.9, 6.7
Yes	47.9	44.6, 51.2	11.4	9.4, 13.4	27.4	24.4, 30.4	13.3	11.2, 15.3
Anxiety								
No	46.8	44.5, 49.1	9.6	8.1, 11.1	36.9	34.8, 39.1	6.7	5.7, 7.6
Yes	48.5	44.9, 52.1	10.4	8.5, 12.4	29.3	26.0, 32.6	11.8	9.8, 13.8
Loneliness								
No	46.2	43.9, 48.6	9.2	7.8, 10.6	38.2	35.9, 40.4	6.4	5.5, 7.3
Yes	49.8	46.3, 53.3	11.4	9.2, 13.6	26.5	23.5, 29.4	12.4	10.2, 14.5

Note: All figures shown in table are population-weighted.

**Table 3:** Multi-nomial logistic regression evaluating the relationship between participants’ mental health status and changes in their alcohol consumption in the past week, COVID-19 Coping Study, April-May 2020, N = 6548.

Mental Health Indicator	Change in Alcohol Consumption (Versus no Change from Usual Consumption)					
	Don’t Usually Do This Activity		Drinking Less than Usual		Drinking More than Usual	
	OR	95% CI	OR	95% CI	OR	95% CI
Depression						
No	1.00 (ref)		1.00 (ref)		1.00 (ref)	
Yes	1.13	0.85, 1.51	1.67	1.23, 2.25	2.65	1.98, 3.55
anxiety						
No	1.00 (ref)		1.00 (ref)		1.00 (ref)	
Yes	1.11	0.82, 1.50	1.27	0.93, 1.73	1.79	1.33, 2.41
Loneliness						
No	1.00 (ref)		1.00 (ref)		1.00 (ref)	
Yes	1.23	0.92, 1.65	1.64	1.21, 2.24	2.42	1.81, 3.25

Note: Models adjusted for age, sex, race, relationship status, education, pre-COVID employment status, history of physician-diagnosed high blood pressure, diabetes, heart disease, asthma, chronic obstructive pulmonary disease, cancer, or any other life-limiting or chronic condition, pre-COVID-19 daily physical activity, pre-COVID-19 weekly alcohol consumption, smoking status, knowing someone diagnosed or with symptoms of COVID-19, and pre-COVID-19 social isolation. All figures shown in the table are population-weighted.

**Table 4:** Number of mental health outcomes participants of the COVID-19 coping study screened positive for in April and May 2020 by self-reported changes in alcohol consumption after the start of the pandemic, N = 6548.

Number of Mental Health Indicators	Population Weighted Percentage (95% CI) of Proportion			
	Don’t Usually Do This Activity (n = 2717)	Drinking Less than Usual (n = 636)	No change in Usual Drinking (n = 2478)	Drinking More than Usual (n = 717)
0 mental health indicators	45.2 (42.4, 48.0)	9.2 (7.4, 10.9)	40.2 (37.5, 42.8)	5.5 (4.4, 6.5)
1 mental health indicator	53.1 (49.2, 57.1)	8.7 (6.4, 11.0)	31.6 (28.0, 35.2)	6.6 (4.9, 8.3)
2 mental health indicators	45.1 (40.5, 49.7)	11.9 (9.1, 14.6)	30.0 (25.7, 34.2)	13.1 (10.4, 15.8)
3 mental health indicators	49.8 (44.1, 55.4)	11.6 (8.2, 15.0)	23.6 (18.8, 28.5)	15.0 (11.3, 18.8)

**Table 5:** Multi-nomial logistic regression evaluating the association between number of mental health outcomes and changes in participants’ alcohol consumption in April/May 2020, COVID-19 Coping Study, N = 6548.

Number of Mental Health Outcomes	Change in Alcohol Consumption (Versus no Change from Usual Consumption)					
	Don’t Usually Do This Activity		Drinking Less than Usual		Drinking More than Usual	
	OR	95% CI	OR	95% CI	OR	95% CI
1 indicator	1.33	0.95, 1.87	1.17	0.81, 1.69	1.32	0.91, 1.94
2 indicators	1.01	0.73, 1.41	1.58	1.09, 2.31	2.67	1.86, 3.83
3 indicators	1.43	0.88, 2.30	2.06	1.28, 3.30	3.83	2.48, 5.92
*p-value for trend*	0.21		0.0007		<0.0001	

Note: Models adjusted for age, sex, race, relationship status, education, pre-COVID employment status, history of physician-diagnosed high blood pressure, diabetes, heart disease, asthma, chronic obstructive pulmonary disease, cancer, or any other life-limiting or chronic condition, pre-COVID-19 daily physical activity, pre-COVID-19 weekly alcohol consumption, smoking status, knowing someone diagnosed or with symptoms of COVID-19, and pre-COVID-19 social isolation. All figures shown in table are population-weighted.

## Data Availability

De-identified data are available upon reasonable request following completion of a data use agreement and paper proposal form (obtained from LCK or JFM), along with appropriate institutional research ethics board approval.
